# Towards a Multidisciplinary Approach to Improve Cattle Health and Production in Uganda

**DOI:** 10.3390/vaccines7040165

**Published:** 2019-10-31

**Authors:** José de la Fuente, Marinela Contreras, Paul D. Kasaija, Christian Gortazar, Jose F. Ruiz-Fons, Rafael Mateo, Fredrick Kabi

**Affiliations:** 1 SaBio, Instituto de Investigación en Recursos Cinegéticos (IREC), Consejo Superior de Investigaciones Científicas (CSIC), Universidad de Castilla-La Mancha (UCLM)-Junta de Comunidades de Castilla-La Mancha (JCCM), Ronda de Toledo s/n, 13005 Ciudad Real, Spain; marinelacr@hotmail.com (M.C.); kpauldavis@gmail.com (P.D.K.); christian.gortazar@uclm.es (C.G.); josefrancisco.ruiz@uclm.es (J.F.R.-F.); rafael.mateo@uclm.es (R.M.); 2 Department of Veterinary Pathobiology, Center for Veterinary Health Sciences, Oklahoma State University, Stillwater, OK 74078, USA; 3 National Livestock Resources Research Institute (NaLIRRI/NARO), Kampala 5704, Uganda; freddykabi@gmail.com

**Keywords:** cattle, tick, vaccine, Uganda, economy

## Abstract

A meeting and course supported by the Vice-Presidency for International Affairs of the Spanish National Research Council (CSIC) and the National Agricultural Research Organization of Uganda (NARO) were held at the National Livestock Resources Research Institute (NaLIRRI) in Nakyesasa, Wakiso, Uganda on September 2–9, 2019. The activities were conducted within the collaboration program between the Institute of Game and Wildlife Research (IREC, CSIC-UCLM-JCCM, Spain) and NARO for the development of vaccines and other interventions for the control of cattle ticks in Uganda.

## 1. Ticks and Tick-Borne Diseases in Uganda: The Need for Novel Vaccine Control Interventions

Tick-borne diseases (TBDs), such as East Coast fever (ECF; caused by *Theileria parva*), babesiosis (caused by *Babesia bigemina*), anaplasmosis (caused by *Anaplasma marginale*), and heartwater (caused by *Ehrlichia ruminantium*), that affect cattle production in Uganda [1] have been proposed, based on archeological evidence from Sub-Saharan Africa, as probable barriers to the early entry of cattle-based economies in these regions [2]. However, current livestock production in Uganda, as in other African countries, that is mainly driven by smallholder farmers constitutes one of the most important contributions to the growth of the country’s economy [3]. 

The costs of controlling tick-borne diseases in Uganda constitute around 80% of the total annual costs to control livestock diseases in the country [4]. For example, ECF can kill annually 30% of newborn calves of indigenous cattle and up to 100% of crossbred or pure cattle [5]. According to the report produced by the presidential Technical Advisory Committee (TAC) on the tick resistance challenge in November 2017 [6], “Uganda’s animal resources-based industry (ARI) combined contributes to about 17% of the national gross domestic product (GDP) (livestock—5%, fisheries—3% and wild animal tourism—8.4%) making it a USD 4.7 billion industry in the country.” They stated that “Ticks are one of the most resilient and harmful pests and parasites in the animal industry. The loss caused by ticks and TBD complex in Uganda alone is estimated at over USD 1.1 billion annually through calf mortality (30% of the calf crop dies), farm disease prevention and control (over 90% of total disease control and treatment costs and 60% of farm input costs), loss in milk (USD 187 million) and meat production (USD 472 million), and blood loss (USD 26 million).” Furthermore, according to this report, “Uganda imports 378,000 liters of acaricides and 83,000 liters of associated drugs causing an annual forex outflow worth over USD 83.3 million.” For example, according to Okello-Onen et al. [3], milk constitutes the most important source of animal protein and accounts for over 16% of the GDP. Indigenous cattle breeds constitute over 95% of the total cattle population, but ticks and TBDs such as ECF cause over 8% mortality in both ranch and pastoral herds and heavily affect their production [3,7]. Consequently, despite a higher cattle growth rate and milk production during heavy rainy seasons, twice-a-week dipping for tick control also increases milk production and preweaning growth rate [3]. 

However, the use of acaricides for the control of cattle tick infestations in Uganda has been generalized without developing strategies considering factors such as tick ecology, epidemiology and endemic stability to TBDs, or the economic impact of TBDs and the different control strategies and practices, therefore causing the emergence of multiacaricide-resistant ticks with a growing impact on cattle health and production [8,9,10,11]. According to TAC’s report [6], tick resistance is widespread and the management of TBDs is chaotic, thus affecting cattle production and productivity, which are essential for socioeconomic growth in Uganda. 

Recently, the Food and Agriculture Organization of the United Nations (FAO), in coordination with the Government of Uganda and stakeholders [12], proposed measures to prevent the economic losses caused by ticks and TBDs in the cattle industry in the country, including (i) the correct use and application of the most appropriate and effective acaricides; (ii) nationwide community and farmer sensitization program on ticks and TBD control; (iii) continuous monitoring of tick populations, pathogen prevalence, and resistance to acaricides; (iv) monitoring, regulating, and limiting wildlife–livestock interactions; and (v) research and development of antitick vaccines.

## 2. Vaccines for the Control of Cattle Tick Infestations in Uganda: An Ongoing Research Project and Future Perspectives

Antitick vaccines constitute an environmentally sound intervention with demonstrated efficacy for the control of cattle ticks and TBDs [13,14]. The first and only commercial vaccines for the control of cattle tick infestations were based on *Rhipicephalus microplus* BM86 or BM95 recombinant antigens and registered in Cuba and Australia in the early 1990s [13,15]. These vaccines are not designed to prevent cattle tick infestations but to reduce tick populations and the prevalence of TBDs by affecting tick feeding, reproduction, and development after ingesting the blood meal with antigen-specific antibodies in immunized animals that interact with and affect protein function [13,14,15]. If the tick vaccines are used consistently for up to three years, tick populations infesting cattle will continuously reduce to below economic levels, which will further translate into reduced frequency of acaricide application [13]. Despite existing challenges and limitations, the evidence supports the development of novel effective vaccines for the control of multispecies tick infestations in cattle [14,16]. 

**Figure 1:**
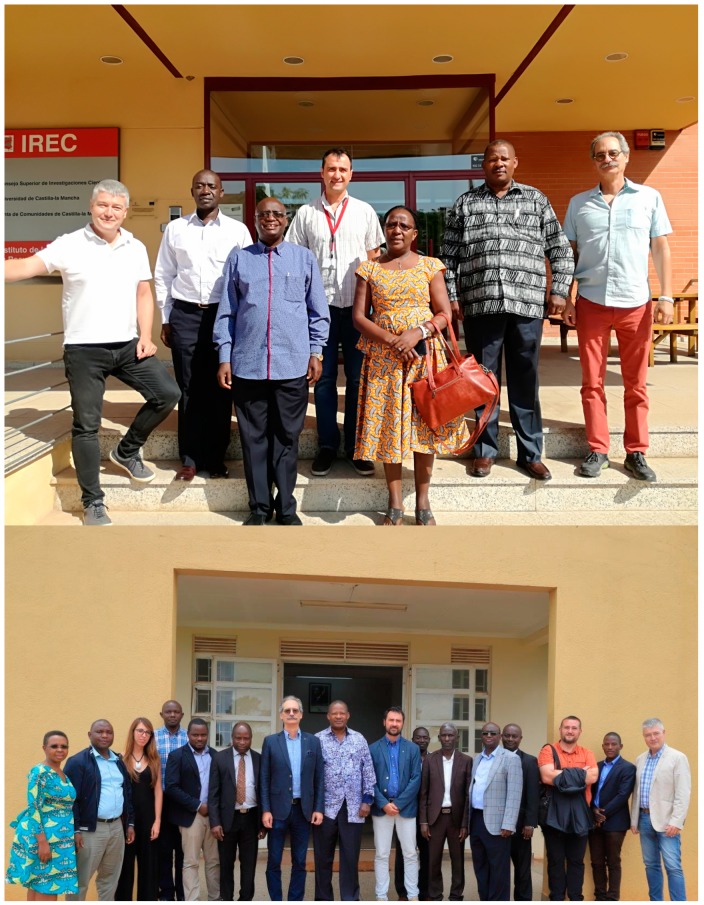
Upper panel: visit of the National Agricultural Research Organization of Uganda (NARO) executives to the Institute of Game and Wildlife Research (IREC) in Ciudad Real, Spain in September 2018. From left to right, C. Gortazar, F. Kabi, J. Rutaisire, R. Mateo, M.T. Kiggundu, A. Agona, and J. de la Fuente. Lower panel: The participants at the meeting between IREC representatives and NARO authorities regarding the development of vaccines for the control of cattle ticks in Uganda. The meeting was held at the National Livestock Resources Research Institute (NaLIRRI) in Wakiso District, Uganda on September 5, 2019. From left to right, I. Kasaija, S. Mugerwa, M. Contreras, M. Dhikusooka, P.D. Kasaija, H. Kirunda, J. de la Fuente, A. Agona, R. Mateo, F. Kabi, Y. Baguma, J. Rutaisire, R. Bangonza, J.J. Ruiz-Fons, J. Mbihayeimaana, and C. Gortazar.

The collaboration between the Institute of Game and Wildlife Research (IREC) and the National Agricultural Research Organization of Uganda (NARO) for the development of vaccines to control cattle tick infestations began with P. D. Kasaija’s arrival at our laboratory at SaBio, IREC to enroll in the Ph.D. program at the University of Castilla, La Mancha in Spain and during the visit of NARO executives to IREC in September 2018 (Figure 1, upper panel). NARO is a statutory body established by an Act of Parliament as the apex body responsible for the coordination of all agricultural research initiatives in the Ugandan agricultural research system. Over the past years, NARO has generated livestock technologies through one of its constituent institutes, the National Livestock Resources Research Institute (NaLIRRI). NaLIRRI’s core mandate is to conduct research on all aspects of livestock, including health, and to provide technical guidance to the government of Uganda. 

To improve livestock production in Uganda, NARO management approved a plan to establish a production facility for a cattle tick vaccine and contacted Professor José de la Fuente at IREC to establish a plan for developing a subolesin (SUB)-based vaccine for the control of cattle tick infestations in Uganda. As stated in the letter by the General Director of NARO, Ambrose Agona, the objectives were (i) understanding the institutional framework that supports successful vaccine production, (ii) identifying the best practices in livestock vaccine production, (iii) identifying infrastructural and human resources requirements for vaccine production, and (iv) establishing a lasting partnership and linkages between NARO and IREC for the development, registration, and production in Uganda of a cattle tick vaccine. 

The tick protective antigen SUB (also known as 4D8) was discovered in 2002 [17], and since then, it has demonstrated protection in vaccines against multiple tick species and other arthropod ectoparasites (recently reviewed by [15,18]). Based on these results, SUB was chosen for cattle tick vaccine development using antigens isolated from local major tick species of *Bos indicus* and crossbred cattle in Uganda, *Rhipicephalus appendiculatus*, *Rhipicephalus decoloratus*, and *Amblyomma variegatum*. Vaccination trials with systemic and oral vaccine formulations are currently ongoing at NaLIRRI with the participation of the Ph.D. student enrolled in the Ph.D. program at the University of Castilla, La Mancha in Spain. This is the first time that vaccines based on SUB antigens are being tried to successfully control multiple cattle tick species. A second meeting between NARO management and IREC representatives was conducted at NaLIRRI in September 5, 2019 to discuss preliminary results of ongoing vaccination trials and future strategies for the control of tick-borne and other diseases affecting cattle health and production in Uganda (Figure 1, lower panel).

## 3. The Course: Building Scientific Capacity in Uganda

**Figure 2:**
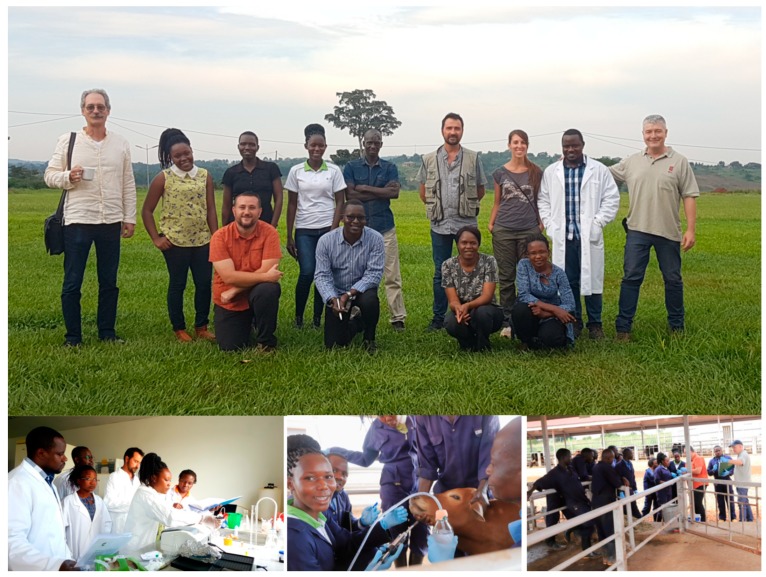
The participants in the course held at NaLIRRI in Wakiso District, Uganda on September 2–9, 2019. J. de la Fuente, M. Contreras, C. Gortazar, F. Ruiz-Fons, and R. Mateo offered the course. The attendees included Senior Research Officers F. Kabi and J. Nakayima; Research Officers R. Alingu, J. Bugeza, M. Dhikusooka, A.L. Mulondo, P. Abila, and G. Nsereko; Principal Lab Technician P. Kasaija (also a Ph.D. student at the University of Castilla, La Mancha in Spain); and Senior Lab Technician S. Kerfwa.

As part of the project, a course was prepared to address the objective of developing the infrastructure and human resources required for the control of ticks and TBDs and vaccine production. The course was intended to build scientific capacity at the NaLIRRI in Uganda at both theoretical and laboratory practical levels and to implement a multidisciplinary approach for the control of infectious diseases affecting humans and animals in Uganda (Figure 2). A special emphasis was put on ticks and tick-borne diseases, multihost shared infections, and other factors affecting human and animal health and livestock production and trade. The program included (i) an introduction to molecular biology and biotechnology and applications for vaccine development by J. de la Fuente and M. Contreras, (ii) epidemiology and control of infections shared with wildlife by C. Gortazar, (iii) the role of wildlife in vector ecology and vector-borne disease epidemiology by F. Ruiz-Fons, and (iv) veterinary toxicology and forensic applications for wildlife conservation by R. Mateo.

## 4. Infections Shared with Cattle: Prospects for Research and Control Interventions

Tick infestation and tick-borne diseases such as anaplasmosis and piroplasmosis are ranked high among the most important cattle diseases for poverty reduction and global trade. However, the list is much longer, including other relevant shared infections such as foot-and-mouth disease (FMD), Rift Valley fever (RVF), brucellosis, and tuberculosis (TB). Often, these shared infections are regarded as “double-burden diseases”, the control of which would benefit both human and animal health and livestock production and trade [19].

One of these priority double-burden diseases is animal TB, caused by *Mycobacterium bovis* and other closely related members of the *Mycobacterium tuberculosis* complex (MTC). It is a typical multihost shared infection present worldwide in a wide range of domestic (e.g., cattle, sheep, goat, and pig) and wild (e.g., buffalo and other wild bovid, wild suid) maintenance hosts [20]. It is known that host species richness correlates with increased community competence to maintain and transmit MTC [21]. However, the specific situation of animal TB in Uganda is only partially known. Animal zoonotic TB is estimated to cause 7.5% of the annual 559/100,000 population total TB cases in Uganda [22]. Regarding cattle, a study in the Mubende District found that 10% of 1576 carcasses inspected had bovine-TB-like lesions, from which MTC was isolated in 12 cases [23], and 14% of 63 cattle herds screened by skin testing in Western Uganda had at least one reactor [24]. In Ugandan wildlife, TB is known to infect both the locally distributed African buffalo (*Syncerus caffer*) and the widespread warthog (*Phacochoerus africanus*) [25]. Therefore, a better understanding of animal TB epidemiology is needed in order to assess the burden of animal TB for human wellbeing, livestock health and production, and wildlife conservation and to eventually discuss future interventions.

The ongoing trials of cattle tick vaccines might provide an opportunity for animal TB control in Uganda. Cattle enrolled in field trials could be tested for TB, possibly using alternatives to the traditional skin test, such as serology, in order to enable single-sampling protocols and facilitate diagnosis in remote regions [26]. Other livestock, particularly sheep and goats, that are likely to be in contact with cattle should also be tested and it would be ideal to include wildlife too. Moreover, further opportunities emerge from the possibility of using heat-inactivated *M. bovis* as an immunostimulant in oral vaccination against ticks [27]. 

Given the key role of cattle in Africa as a safety net and a ladder out of poverty, it is important to maximize the contribution of research activities on livestock disease control to global health, wealth, equity, and sustainability [19].

## 5. Ecology and Epidemiology of Vector-Borne Diseases in Uganda

In Uganda, several arthropods are vectors of pathogens of medical and/or veterinary relevance, with mosquitoes and tsetse flies ranking highest as vectors for human pathogens and ticks having a massive impact on livestock production. 

Tick species differ in their host selection plasticity, with highly specialized one-host ticks such as *R. decoloratus* being less plastic than the three-host ticks *A. variegatum* and *R. appendiculatus*. However, all three species of ticks of relevance for cattle in Uganda can parasitize wild mammals, such as wild bovids, suids, lagomorphs, hedgehogs, carnivores, and small rodents, and a high number of wild bird species [28,29], as well as other domestic animals [30]. Despite its small size, Uganda is among the most biodiverse countries on the African continent. It hosts half of the 2000 bird species of Africa and around 345 mammalian species, making their ecosystems highly complex and, consequently, the epidemiological scenarios for wildlife–livestock shared pathogens. Wildlife conservation can, in particular areas, constitute a matter of conflict with farmers, not only in terms of competition for shared resources but also in terms of livestock and wildlife health [31,32]. 

Oura et al. [33] highlighted the relevance of particular Ugandan wildlife species in the maintenance of tick-borne pathogens shared with livestock, showing that wild ruminants are able to maintain tick-borne pathogens shared with cattle and also to act as reservoirs of pathogens that may in the future emerge as novel diseases of cattle. Uganda suffers from an important gap in the existing information on the tick species that parasitize wildlife and a proper characterization of the scenarios of wildlife–livestock interaction that effectively result in tick and tick-borne pathogen exchange.

Consequently, Uganda faces a potentially increasing wildlife–human conflict to preserve the biodiversity that attracts tourists and supports the services sector of the country, which accounts for 50% of the GDP. Fighting ticks of cattle with acaricides and antitick vaccines would aid but not solve the conflict, which could only be solved by increasing scientific knowledge with a One Health approach that includes humans, livestock, wildlife, and ecosystems together to understand the ecology of ticks and tick-borne pathogens. This would allow designing effective and environmentally friendly prevention and control strategies in the future to control tick infestations and TBDs of cattle in Uganda. This was one of the main topics of the course at NaLIRRI, which was intended to build scientific capacity and increase concern about wildlife as relevant in the life cycle of ticks and tick-borne pathogens in Uganda.

## 6. Veterinary Toxicology: Implications for Tick Control and Cattle Production in Uganda

Exposure to cattle ticks or acaricides such as organophosphates account for pediatric poisonings in rural Uganda [34] and toxic effects in both workers and cattle [35,36]. Organophosphates and α2 agonists, two chemical families that include most of the current acaricides, are frequently used for self-poisonings in Uganda [37,38]. This highlights the risk that comes with the uncontrolled availability in communities of highly toxic pesticides that can be used for other nonlegal purposes, such as wildlife poisoning [39], or even criminal purposes against people [40].

The uncontrolled use of this acaricide may lead to environmental damage in aquatic ecosystems, acaricide resistance in ticks, and a possible exacerbation of TBDs [41]. Although the persistence of most of the acaricides and pesticides currently used in livestock is much lower than that of the organochlorine pesticides, their withdrawal period before obtaining animal-derived products for food must be strictly observed [42]. In addition, the presence of current and legacy pesticides must be monitored in livestock products such as milk and meat to avoid risks to human consumers [43,44].

Therefore, the correct use and application of the most appropriate and effective acaricides, which was recently proposed as one of the key measures to prevent economic losses caused by ticks and TBDs in the cattle industry in Uganda [12], should be included in combination with other control interventions such as antitick vaccines for the effective and sustainable control of TBDs.

## 7. Conclusions and Future Directions

The control of ticks and TBDs constitutes a priority for the Government of Uganda to promote economic growth in the country. This challenge requires a multidisciplinary approach to combine different control measures that range from cattle management and the rational use of acaricides to antitick vaccine development and application. Collaboration between research institutions leading research in these areas with those located in the most affected countries is necessary to achieve these goals. The ongoing collaboration project between IREC and NARO is focused on developing and implementing the production and application of SUB-based vaccines for the control of cattle tick infestations in combination with other control interventions. Controlled pen vaccination trials are currently ongoing. Future directions will include (i) characterization of protective immune response in cattle and vaccine antigen design, (ii) development and evaluation of oral vaccine formulations, (iii) building of vaccine production facilities at NARO, (iv) field trials with effective antitick vaccine formation, (v) characterization of animal TB epidemiology and future control options, (vi) study of the ecology and epidemiology of vector-borne diseases for effective control and prevention, and (vii) application of veterinary toxicology approaches to reduce the detrimental effects of acaricides and other compounds on human and animal health and cattle production.
